# Underexpression of miR-34a in Hepatocellular Carcinoma and Its Contribution towards Enhancement of Proliferating Inhibitory Effects of Agents Targeting c-MET

**DOI:** 10.1371/journal.pone.0061054

**Published:** 2013-04-10

**Authors:** Yiwu Dang, Dianzhong Luo, Minhua Rong, Gang Chen

**Affiliations:** 1 Department of Pathology, The First Affiliated Hospital of Guangxi Medical University, Nanning, Guangxi Zhuang Autonomous Region, People's Republic of China; 2 Research Department, The Affiliated Cancer Hospital of Guangxi Medical University, Nanning, Guangxi Zhuang Autonomous Region, People's Republic of China; The University of Hong Kong, China

## Abstract

Aberrant expression of microRNA-34a (miR-34a) has been reported to be involved in the tumorigenesis and progression of various classes of malignancies. However, its role in hepatocellular carcinoma (HCC) has not been completely clarified. In the current study, we have investigated the clinical significance and the *in vitro* contribution of miR-34a on biological functions of human HCCs. miR-34a expression in eighty-three cases of HCC formalin-fixed paraffin-embedded (FFPE) tissues decreased significantly compared to that in the adjacent liver tissues (*P*<0.01), as detected by real-time quantitative RT-PCR (RT-qPCR). miR-34a expression in the groups of TNM stage I and II, without metastasis and without portal vein tumor embolus, was significantly higher than that of their corresponding groups (*P*<0.05). In functional experiments, miR-34a mimic suppressed cell growth, migration and invasion, meanwhile it increased cellular apoptosis and caspase activity in HCC cells. miR-34a mimic also reduced phospho-ERK1/2 and phospho-stat5 signaling. In addition, miR-34a mimic enhanced the effect of cell proliferation inhibition and caspase activity induction of agents targeting c-MET (siRNAs and small molecular inhibitor su11274). In conclusion, miR-34a may act as a tumor suppressor miRNA of HCC. The strategies to increase miR-34a level might be a critical targeted therapy for HCC in future.

## Introduction

Studies have shown that aberrant microRNAs (miRNAs) expression is correlated with the development and progression of cancer, thus miRNAs could be used as biomarkers for diagnosis and prognosis of cancer. On the other hand, the miRNAs can have oncogenic or tumor suppressor activities, so miRNAs are emerging as vital targets for cancer molecular therapies [1]. Hepatocellular carcinoma (HCC) ranks in prevalence and mortality among the top 10 cancers all over the world. The estimated number of new cases of HCC had risen to 564, 300 and 548, 600 patients with HCC had died, representing 97.2% of persons with this diagnosis [2]. The development and progression of HCC in humans is a multi-step, long-term process, characterized by the progressive accumulation of genetic and epigenetic alterations associated with sequential evolution of morphologically distinct stages culminating in the formation of fully developed HCC. Many reports have highlighted on investigating genes and proteins underlying the development and progression of HCC [3], [4], [5], [6], [7], [8], [9], [10], however, their sensitivity and specificity remain suboptimal. Therefore, the identification of new biomarkers is urgently needed in order to understand the events causing hepatocarcinogenesis, also to relate various phenotypes in clinical features and prognosis and, more importantly, to predict response possibilities to therapeutic approaches. Extensive profiling studies over the past several years have shown that various miRNAs are differentially expressed in HCC [11], [12]. Nevertheless, the involvement of miRNAs in hepatocarcinogenesis and progression of HCC remains to be clarified. Among all the HCC-related miRNAs, contradictory relationship between miR-34a levels and HCC was reported [13], [14]. Furthermore, the relationship between the miR-34a expression and clinicopathological parameters in HCC was not fully understood. In the present study, we investigated the expression of miRNA-34a in HCC and their matched adjacent noncancerous liver tissues in 83 cases of formalin-fixed paraffin-embedded (FFPE) surgically resected samples, using real time quantitative RT-PCR (RT-qPCR). Additionally, we performed *in vitro* experiments to study the effect of miR-34a on the cell growth, apoptosis, caspase-3/7 activity, migration and invasion in HCC cell lines. c-MET is a proved target gene of miR-34a [14], [15] and c-MET inhibitor demonstrated a manageable safety profile and preliminary antitumor activity in patients with HCC and Child-Pugh A or B cirrhosis [16], hence we have for the first time investigated the combinatorial effect of miR-34a mimic and c-MET targeting agents (c-MET siRNAs or c-MET kinase inhibitor su11274) in HCC cells.

## Results

### miR-34a expression in HCC FFPE tissues and its clinicopathological significance

**Figure 1:**
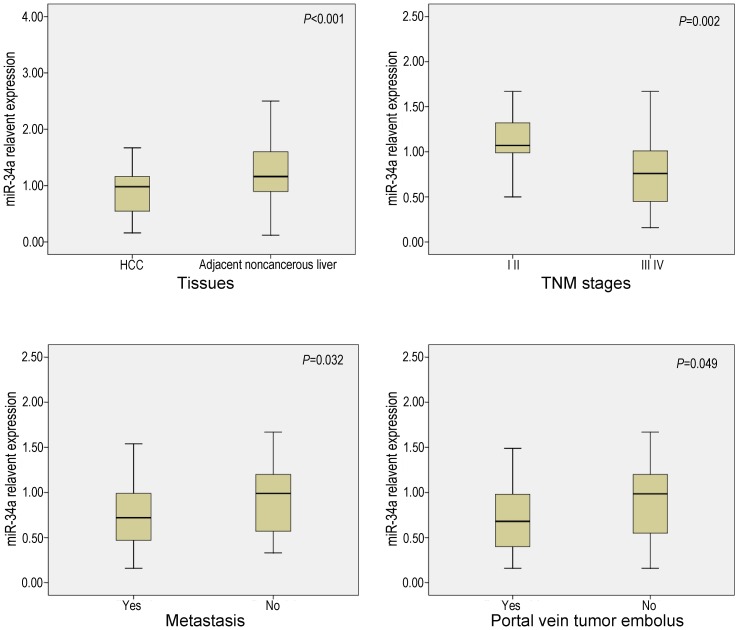
Relationship between miR-34a expression and clinicopathological parameters. miR-34a expression was determined using real time RT-qPCR.

The relative expression of miR-34a in HCC tissues was significantly lower than that of their matched adjacent noncancerous liver tissues (*P*<0.01). The expression of miR-34a in the tissues in clinical TNM III and IV stages was significantly lower than that in I and II stages. Furthermore, in the group with metastasis, miR-34a expression was down-regulated compared to the group without metastasis (*P*<0.05). When studied the relationship between miR-34a expression and other clinicopathological parameters, we found that miR-34a level was correlated with the status of portal vein tumor embolus. miR-34a level was lower in the cases with portal vein tumor embolus than those without (*P*<0.05, Table 1, Figure 1). miR-34a level was also found lower in males than in females. The miR-34a however had no correlation with other features, such as age, histological differentiation grades, cirrhosis, plasma AFP levels, tumor capsular infiltration, number of the tumor nodes or tumor sizes.

**Table 1:** Correlation between the expression of miR-34a and Clinicopathological Parameters in HCC 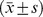.

Clinicopathological Parameters		n	miR-34a relavant expression		
			*2^−Δcq^*	*t*	*P*
Tissue	HCC	83	0.8767±0.4207	9.641▵	0.000
	Adjacent noncancerous liver	83	1.2586±0.5489		
Age	≥50	40	0.8107±0.3862	1.386	0.170
	<50	43	0.9381±0.4461		
Gender	Male	67	0.8299±0.4131	2.122	0.037
	Female	16	1.0731±0.4072		
Differentiation	Well	5	0.7760±0.2621	*F* = 2.075*	0.133
	Moderately	53	0.9540±0.4298		
	Poorly	25	0.7932±0.3720		
Clinical TNM stage	I & II	22	1.1286±0.4160	3.374	0.002
	III & IV	61	0.7859±0.3869		
Metastasis	Yes	34	0.7585±0.3934	2.181	0.032
	No	49	0.9588±0.4233		
With cirrhosis	Yes	41	0.8490±0.4100	0.591	0.556
	No	42	0.9038±0.4341		
AFP (µg/L)	≥400	45	0.9284±0.4612	1.004	0.319
	<400	38	0.8225±0.4094		
Portal vein tumor embolus	Yes	21	0.7329±0.4068	1.994	0.049
	No	62	0.9255±0.4173		
Tumor capsular infiltration	No capsular or capsular infiltration	42	0.8895±0.3936	0.278	0.781
	No capsular infiltration	41	0.8637±0.4513		
Tumor nodes	Multi	35	0.7957±0.3867	1.510	0.135
	Single	48	0.9358±0.4387		
tumor diameter(cm)	≥5	67	0.8496±0.4246	1.170	0.245
	<5	15	0.9907±0.4103		
Cirrhotic adjacent liver	Yes	41	1.1649±0.6081	1.549	0.127
	No	42	1.3500±0.4738		

▵ Paired samples *t*-test was performed.

* One-way Analysis of Variance (ANOVA) test was performed.

### Effect of miR-34a on malignant phenotype in HCC cells

**Figure 2:**
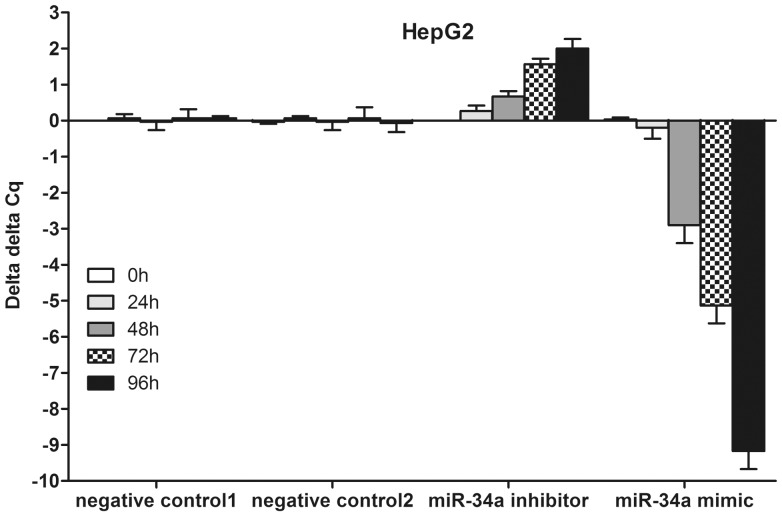
Transfection efficiency of miR-34a inhibitor and miR-34a mimic in HepG2 cells. HepG2 cells (2.5×10^4^ cells per well using 6-well-plate) were transfected with miR-34a inhibitor, miR-34a mimic and their negative controls up to 96 h. Expression of miR-34a was detected using real time RT-qPCR and delta delta Cq was calculated.

**Figure 3:**
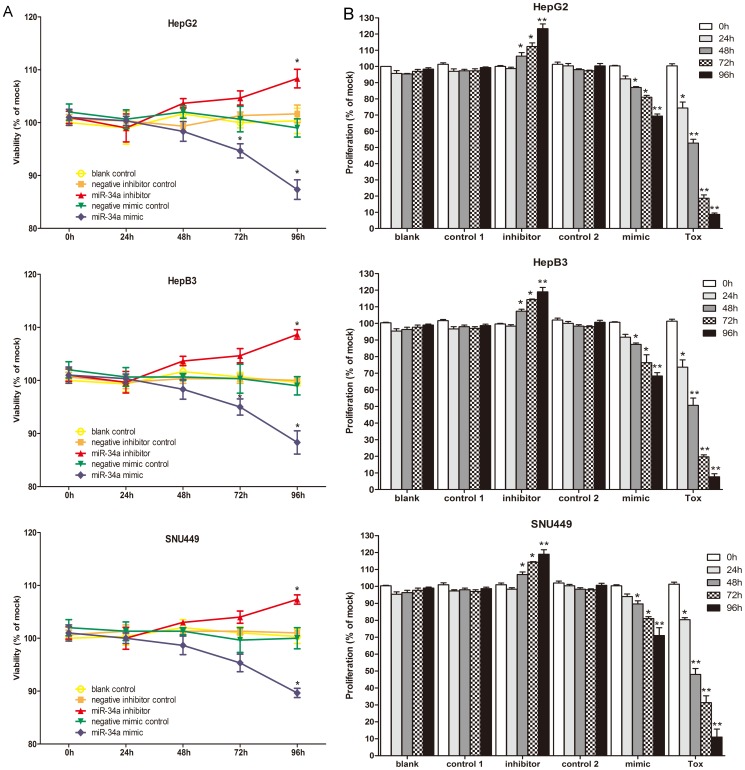
Effect of miR-34a on cell growth in HCC cells. HepG2, HepB3 and SNU449 cells (2.5×10^3^ cells per well in 96-well-plate) were cultured for one day and then transfected with miR-34a inhibitor, miR-34a mimic and their controls (200 nM). (A) Time dependent effect detected by CellTiter-Blue Cell Viability assay. (B) Cell proliferation assessed with MTS assay. * *P*<0.05, ** *P*<0.01, compared to blank and negative controls at the same time point. TOX: TOX positive transfection control.

**Figure 4:**
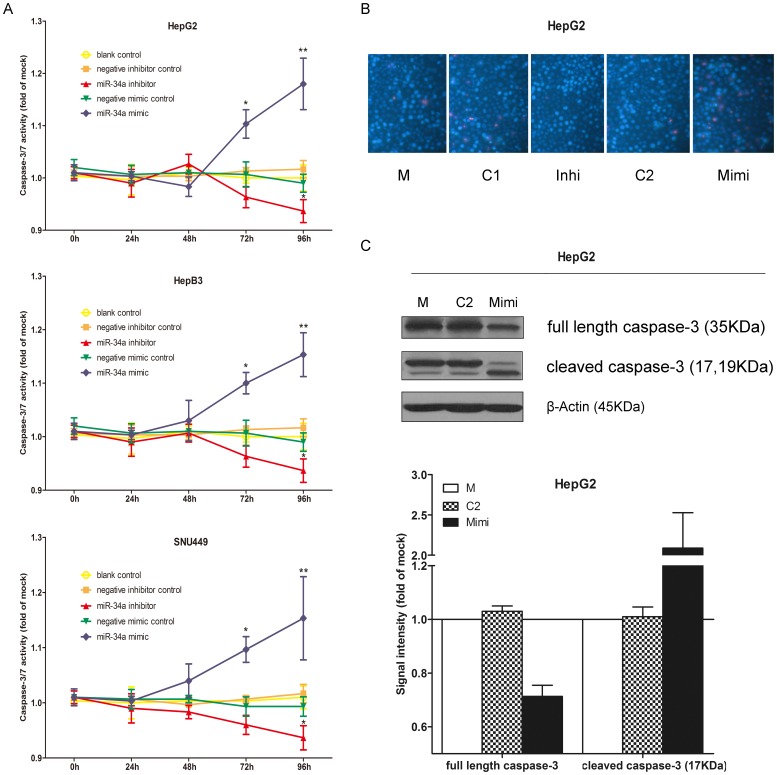
Effect of miR-34a on apoptosis in HCC cells. HepG2, HepB3 and SNU449 cells were treated as in Figure 2. (A) Caspase-3/7 activity was detected using Apo-ONE Homogeneous Caspase-3/7 Assay. * *P*<0.05, ** *P*<0.01, compared to blank and negative controls at the same time point. (B) Hoechst 33342/PI double fluorescent chromatin staining with different transfections for 96 hrs in HepG2 cells (×200). (C) Western blot and signal intensity of the bands. Cells were treated for 96 h. Antibodies include: full length caspase-3, cleaved caspase-3 and β-actin. M: mock control; C1: Negative control for miRNA inhibitor; Inhi: miR-34a inhibitor; C2: Negative control for miRNA mimic; Mimi: miR-34a mimic.

**Figure 5:**
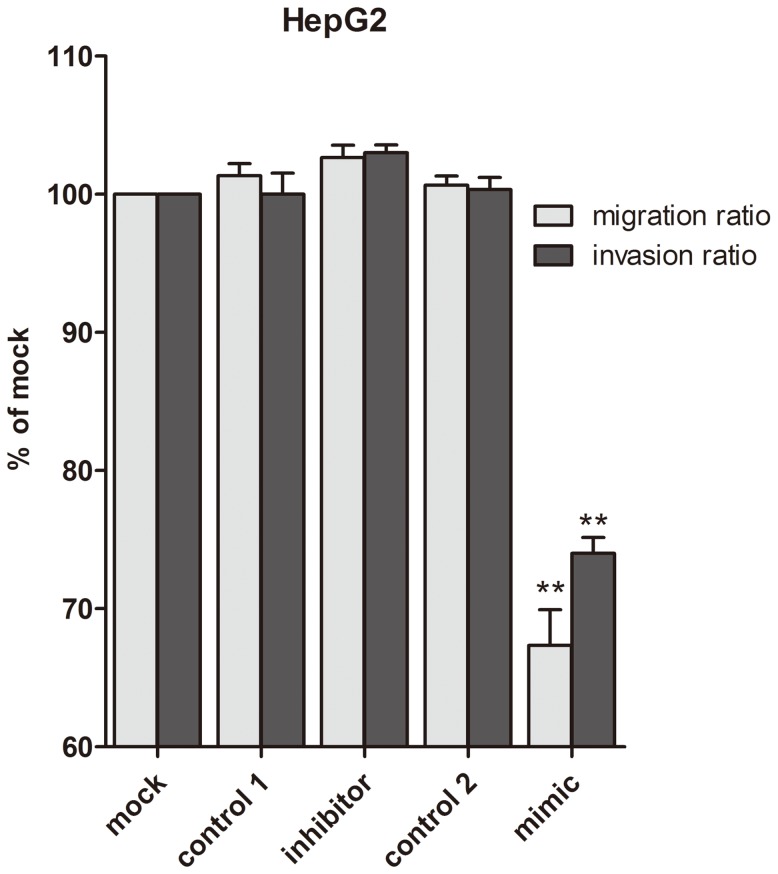
Effect of miR-34a inhibitor and miR-34a mimic on cell migration and invasion in HepG2 cells. HepG2 cells (5×10^4^ cells per well using 6-well-plate) were cultured to 50% confluent and transfected with the miR-34a inhibitor, miR-34a mimic and the controls. The cells were incubated in serum-free medium for another one day 96 h post transfection. Then the cells were trypsinized and added (5×10^4^ cells) to the chamber (6.5 mm in diameter, 8 µm pore size) for both migration and invasion assays. migrating and invasive cells were fixed, stained, counted and the ratio of cell migration and cell invasion was calculated.

**Figure 6:**
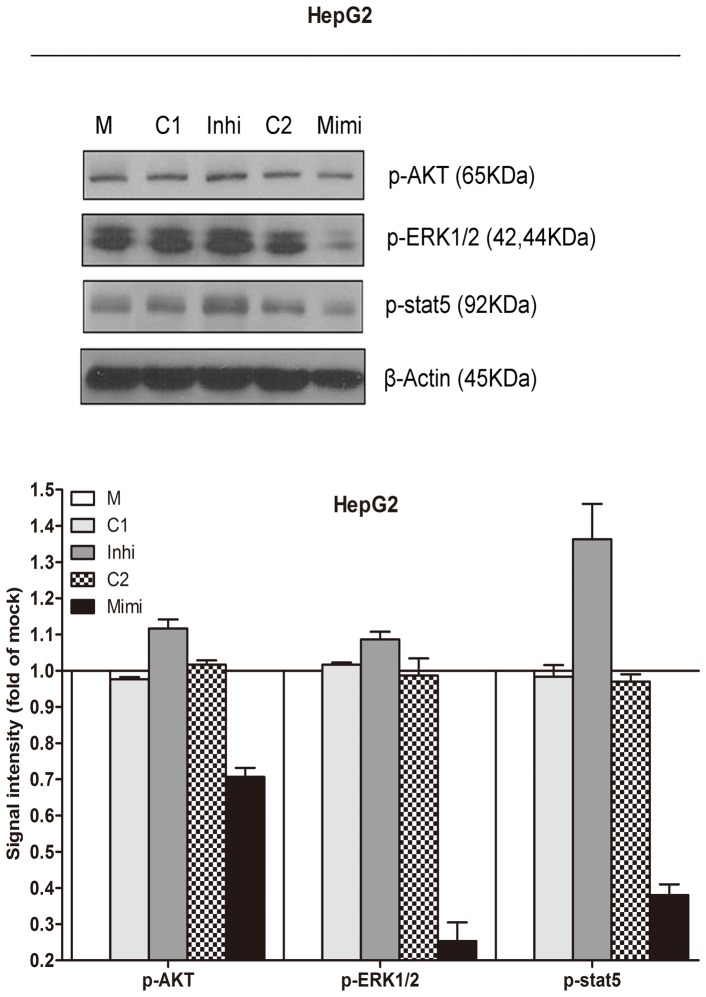
Effect of miR-34a on downstream pathway signals in HCC cells. Western blot and signal intensity of the bands. Antibodies include: phospho-AKT (p-AKT), p-ERK1/2, p-stat5 and β-actin. M: mock control; C1: Negative control for miRNA inhibitor; Inhi: miR-34a inhibitor; C2: Negative control for miRNA mimic; Mimi: miR-34a mimic.

Transfection efficiency was monitored using real time RT-qPCR (Figure 2). The effect of miR-34a on cell viability was detected using a fluorimetric resorufin viability assay. With the miR-34a inhibitor, cell viability was slightly increased in HepG2, HepB3 and SNU449 cells 96 h post-transfection compared to negative controls, however the difference was not significant. After transfection with the miR-34a mimic, a moderate decreasing in viability was noted at the 96 h in all the three cell lines (Figure 3A). To verify these results, the effect on cell proliferation was assessed using a MTS tetrazolium assay (Figure 3B) and likewise by microscopic counting of viable (Hoechst 33342 positive/PI negative) cells (Figure 4), which both largely mirrored the fluorimetric resorufin viability assay results. To determine whether miR-34a is able to influence apoptosis, the CellTiter-Blue assay was multiplexed with a fluorescent caspase-3/7 assay. The results showed that with the miR-34a inhibitor, caspase-3/7 activity was slightly downregulated than the negative controls, but contained no significant difference. However, with the miR-34a mimic, caspase- 3/7 activity significantly enhanced at the 72 and 96 h post-transfection in all three cell lines (Figure 4A). The effect on apoptosis was confirmed microscopically by Hoechst 33342 and PI double fluorescent staining (Figure 4B) and also by the detection of cleaved caspase-3 with western blot (Figure 4C). Next, we evaluated the role of miR-34a function on the migration and invasion of HepG2 cells. The miR-34a inhibitor had little effect on the migration and invasion activity. The miR-34a mimic led to a moderate decreased migration and invasion rate in HepG2 (Figure 5). To investigate the contribution of miR-34a in the regulation of cellular signaling, we examined by western blot the signaling of AKT, ERK and stat pathways, which are related to cell survival, apoptosis, migration and invasion. These pathways were influenced little with miR-34a inhibitor transfection, however, the phospho-ERK1/2 and phospho-stat5 were down-regulated by miR-34a mimic 96 h post-transfection (Figure 6).

### miR-34a mimic enhanced the cell proliferation inhibitory effect of c-MET siRNA and of su11274

**Figure 7:**
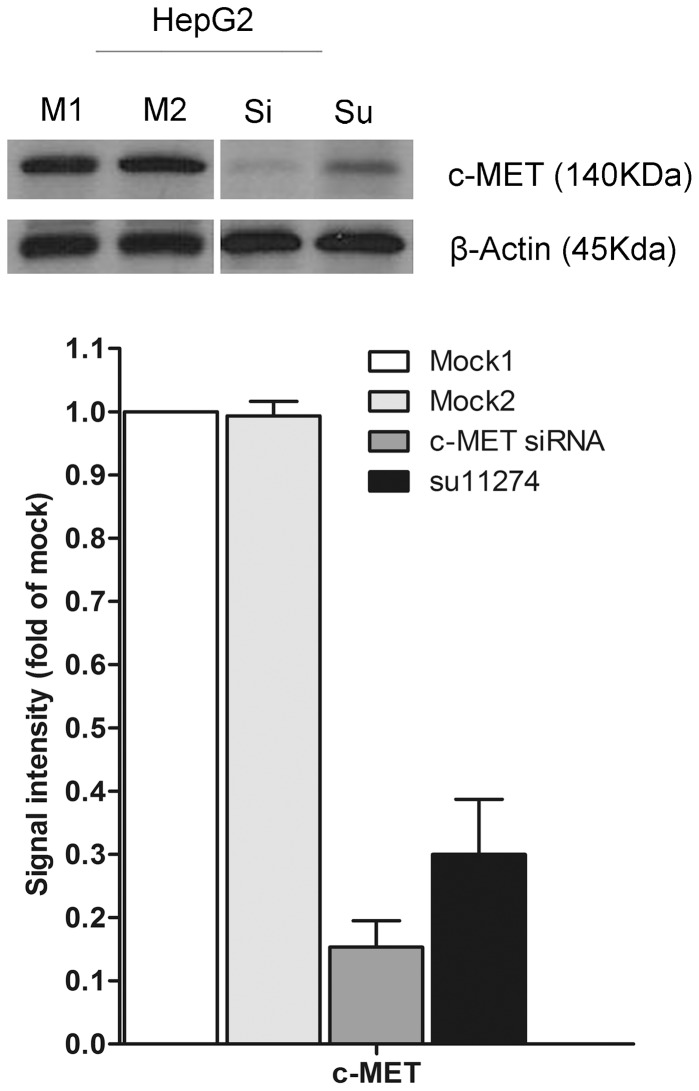
Protein level of c-MET after treatment of siRNAs or small molecular inhibitor su11274. HepG2 cells (2.5×10^4^ cells per well using 24-well-plate) were transfected with c-MET siRNAs or treated with c-MET small molecular inhibitor su11274 for 96 h. c-MET protein level was determined using western blot. c-MET and β-actin western blot signals were quantified, and the c-MET signal intensity relative to the β-actin was calculated. These values are represented by the bar graph. M1:Mock1, mock control for siRNA containing only transfection reagent; M2: Mock2, mock control for su11274 with only 0.1% DMSO. Si: c-MET siRNAs. Su: su11274.

**Figure 8:**
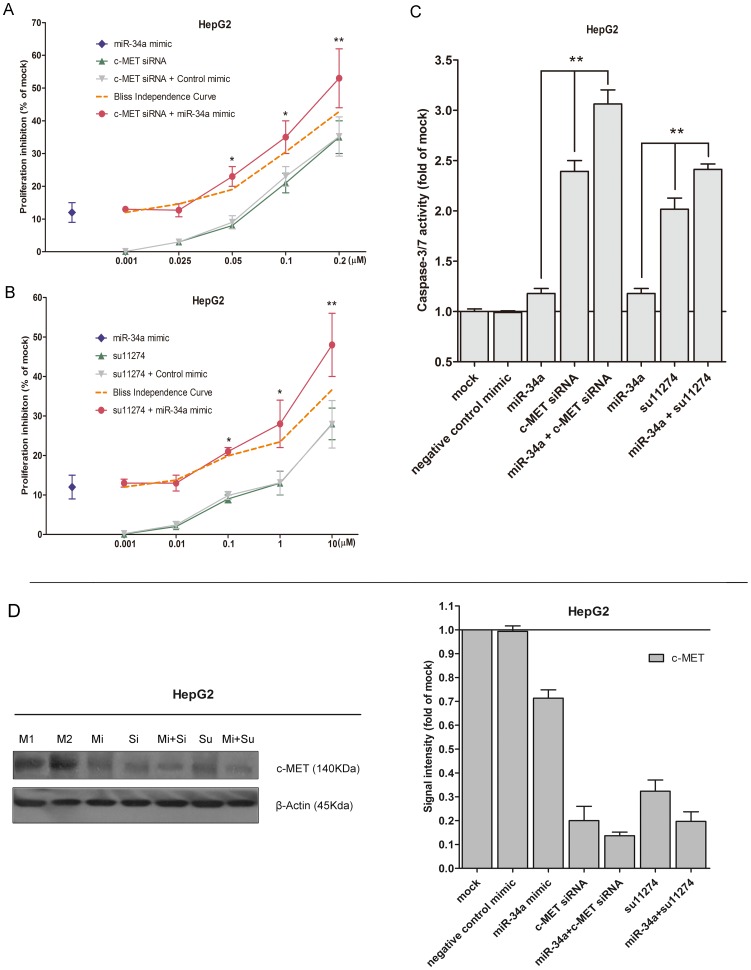
miR-34a enhanced the growth inhibitory effect of c-MET targeting agents in HCC HepG2 cells. HepG2 cells (2.5×10^3^ cells per well in 96-well-plate) were treated with miR-34a mimic with c-MET siRNA (A) or su11274 (B). MTS was performed as Figure 2 and the proliferation inhibition rate was calculated. Bliss independence criterion was performed to calculate the theoretical additive effect. (C) Caspase-3/7 activity. * *P*<0.05, ** *P*<0.01, compared to agent alone. (D) c-MET protein level with single or dual treatments detected by western blot. Antibodies include: c-MET and β-actin.

It was reported that c-MET is a target gene of miR-34a [14], [15], We desired to explore the combinatorial effect of the miR-34a mimic and agents targeting c-MET (siRNA or small molecular inhibitor, su11274), using the colorimetric MTS formazan proliferation assay. Both of the c-MET siRNAs and inhibitor su11274 could downregulate c-MET protein expression up to 70% (Figure 7). The inhibition of cell proliferation and induction of caspase activity were much stronger with miR-34a mimic in combination with c-MET siRNA or su11274, compared to single drug or single miR-34a mimic in HepG2 cells (Figure 8A, Figure 8B, Figure 8C). By western blot, the down-regulation of c-MET protein expression was also enhanced with the dual treatment, compared to the single treatment (Figure 8D). However, the proliferation curve of the combinatorial treatment was not significantly higher than the Bliss independence curve, which indicated an additive effect. To verify the additive or synergistic nature of combining c-MET targeted agents with the miR-34a mimic, a CI was calculated [17]. This unambiguously showed that the effect is entirely additive, since the CI was not below one (Figure S1 and Figure S2).

## Discussion

miR-34a was reported to be down-regulated in rat liver during hepatocarcinogenesis induced by a methyl-deficient diet, which is relevant to the hepatocarcinogenesis in humans associated with viral hepatitis C and B infections, alcohol exposure and metabolic liver diseases [18]. Contradictorily, miR-34a was found to be consistently up-regulated in the HCCs as compared to the non-neoplastic liver tissues in a chemical-induced HCC F344 rat model [19]. The circulating miR-34a level was also revealed to be gradually increased with the progress of hepatocarcinogenesis in the same rat model [19]. In human HCCs, there were also discrepant reports on the expression of miR-34a. Pineau et al [13] observed by microarray that miR-34a increased in HCC and was linked to disease progression from normal liver through cirrhosis to full-blown HCC. On the contrary, Li et al [14] reported that down-regulation of miR-34a expression was highly significant in 19 of 25 (76%) human HCC tissues compared with adjacent normal tissues, using real time RT-qPCR. Different source of the samples, various assays might partly explain the discordance of different results. In the current study, the result with real time RT-qPCR confirmed the previous report from Li, et al [14], in a larger size of patients of 83 cases, which showed that HCC had lower miR-34a level than the adjacent non-cancerous liver tissues. The underexpression of miR-34a in HCC indicates that miR-34a may play a critical role in the hepatocarcinogenesis, as a tumor suppressor miRNA.

Concerning the relationship between miR-34a expression and clinicopathological features, in the present study, miR-34a expression downregulated in the group with metastasis compared to the group with no metastasis, which is consistent with Li et al [14]. Moreover, we found that miR-34a level was correlated with the status of portal vein tumor embolus. miR-34a expression decreased in the cases that the tumor cells invaded into the portal vein. Generally, the status of portal vein tumor embolus reflects tumor invasion and metastasis. Thus, the result in current study reveals an obvious relation between miR-34a and the migration, invasion and metastasis of HCC. When studied the relationship between miR-34a expression and clinical TNM stages, we found that the downregulation of miR-34a was related to the progression of HCC. Hence it may be valuable to examine miR-34a expression for the clinical prediction of metastasis and progression of HCC. Interestingly, we also found that miR-34a level was lower in males than females, which had never been reported previously. Li et al [14] studied the expression of miR-34a of 25 cases, with only 3 females included. In our current study, more than 5 times of the female cases were available (16 cases). However, further substantiation in a larger cohort is warranted to investigate the relationship between miR-34a level and gender.

miR-34a was also studied functionally *in vitro* in HCC cells. Li et al [14] transfected miR-34a duplex oligoribonucleotides into HepG2 cells up to 48 h and cell proliferation was determined using Cell Counting Kit-8. The results showed that the ectopic expression of miR-34a had no significant inhibition of cell proliferation [14]. Cheng et al [20] transfected the chemically synthesized pre-has-miR-34a-PGCSIL-GFP into the same HCC cell line HepG2. miR-34a showed the discordant effect on cell proliferation compared to what Li et al [14] reported. The transfection of pre-has-miR-34a-PGCSIL-GFP caused a remarkable inhibition of cell proliferation 72 h post-transfection. Additionally, Cheng et al [20] also found that the pre-has-miR-34a-PGCSIL-GFP induced an accumulation of HepG2 cells in G1 phase and reduction of cells in S and G2 phase. In the current study, we transfected miR-34a inhibitor and mimic by combiMAGnetofection into 3 different HCC cell lines. The cell growth was monitored by three independent assays: CellTiter96 AQueous One Solution Cell Proliferation Assay, CellTiter-Blue Cell Viability Assay and Hoechst 33342/PI double fluorescent chromatin staining, respectively. The results of the three methods were in agreement with each other and supported the observation of Cheng et al [20]. With miR-34a mimic, the cell growth inhibitory effect showed a time dependent manner and the cell growth was significantly inhibited 96 h post-transfection. The influence of miR-34a on apoptosis in HepG2 cells was also explored previously by two groups. After transfection of miR-34a duplex oligoribonucleotides for 48 h, Li et al [14] subjected HepG2 cells to DNA content analysis by flow cytometry. There was no significant change of sub-G1 DNA content in HepG2 cells, which was indicative of no effect of apoptosis by miR-34a. In line with this, Cheng et al [20] did not find that the overexpression of miR-34a altered apoptosis in HepG2 cells quantified by Annexin V staining. However, distinct results were observed in the current study. Hoechst 33342/PI double fluerenscent staining, caspase-3/7 activity assay and detection of cleaved caspase-3were performed to test the effect of miR-34a on apoptosis in HCC HepG2 cells. miR-34a mimic induced the apoptosis and caspase activity from 72 h post-transfection and the influence reached the highest summit at 96 h post-transfection. The different methods to re-express miR-34a (miR-34a duplex oligoribonucleotides vs synthesized pre-has-miR-34a-PGCSIL-GFP vs miR-34a mimic) and different time points of transfections (48 vs 72 vs 96 h) might partially explain the discreapancies between other reports and the current results. miR-34a was noted to inhibit migration and invasion in HCC cells [14], [20], which was also confirmed in the present study. Hence, our results suggested that miR-34a could not only inhibit cell growth, migration and invasion, but also induce apoptosis.

The mechanisms of miR-34a inhibiting cell growth, metastasis and inducing apoptosis could be correlated to different networks between miR-34a and other target genes. miR-34a is a direct transcriptional target of p53, which is a transcription factor that coordinates cellular responses to stresses such as DNA damage and oncogene activation. When p53 was induced, it alters the expression of a large set of target genes leading to cell-cycle arrest, apoptosis, increased DNA repair, and/or inhibition of angiogenesis. miR-34a is suggested to be an important component of the p53 tumor suppressor network [21]. Other targets of miR-34a involved in cell cycle, cell growth, invasion and apoptosis including Cyclin D1, Cyclin E2, E2F, B-cell CLL/lymphoma 2 (BCL2), CCNE2, CCND1, microtubule actin cross-linking factor 1 (MACF1), cyclin-dependent kinase 6 (CDK6), CDK4, Lamin-A/C, microtubuleactin cross-linking factor, tubulin a-1B chain, Glial fibrillary acidic protein (GFAP), Tropomyosin α-4 chain (TPM4), chaperone protein Endoplasmin (HSP90B1), Lamin-A/C (LMNA), Aldehyde dehydrogenase (ALDH2), Leucine-rich repeat-containing protein (LOC100129335), Cathepsin D, baculoviral IAP repeat-containing 3 (BIRC3), decoy receptor 3 (DcR3 also known as TNFRSF6B) and c-MET [14], [20], [21]. Further exploration is needed to investigate target genes of miR-34a of HCC cells.

Higher expression of c-MET in tumor tissue can lead to scattering, angiogenesis, proliferation, enhanced cell motility, invasion, and eventually, metastasis [22], [23], [24], [25], [26]. c-MET inhibitors have entered the clinical studies of HCC. For instance, an oral, selective, c-MET inhibitor, tivantinib (ARQ 197) has also shown a manageable safety profile and preliminary antitumor activity in patients with HCC in a Phase-1b study [16]. c-MET is a known target gene of miR-34a [14], [15]. The transfection of miR-34a into HepG2 cell resulted in the c-MET gene silencing [14], which was also confirmed in the present study (Figure 8D). miR-34a was also shown to reduce the phosphorylation of ERK1/2, a key factor influencing the tumor growth, migration and invasion [14]. Here, immunoblotting showed a consistent down-regulation of phospho-ERK1/2 with the founding of Li et al. [14]. We also discovered that another downstream proliferative/survival and anti-apoptotic signal pathway phospho-stat5 was suppressed.

The next question was therefore whether simultaneous inhibition of c-MET, by RNAi or kinase inhibitor, together with miR-34a, would result in increased biological effects. The addition of miR-34a mimic to c-MET siRNAs, led to an greater effect on cell growth inhibition and apoptosis induction. The combinatorial effect was additive, not synergistic, by both Bliss independence criterion and Biosoft CalcuSyn program. Similar combinatorial effect was observed when the miR-34a mimic was added to the c-MET small molecular inhibitor su1174. Thus, we demonstrated that miR-34a mimic enhanced the cell proliferation inhibitory effect by c-MET siRNA and c-MET inhibitor su11274.

Together with previous reports, the current observations strongly proved that miR-34a is a tumor suppressor miRNA that plays a vital role in the oncogenesis and progression of HCC, by targeting multiple pathways. On the other hand, cell growth and invasion inhibition, as well as apoptosis induction by miR-34a mimic appear of great relevance due to its possible therapeutic role. The use of miR-34a mimic, together with other treatments, for instance, agents targeting c-MET pathway, might thus be a promising approach to HCC therapies in the future, to be explored *in vivo* and in clinic.

## Materials and Methods

### Patients

The ages of the 83 cases of HCC patients ranged from 29 to 81 years old, with a mean of 52 years. Clinicopathological information was obtained from medical records and summarized in Table 1. The corresponding paraneoplastic tissues were taken at least 2 cm from the cancerous node. All cases were initial hepatectomies and randomly chosen from the hepatectomies performed over a 1–2 year span in the First Affiliated Hospital, Guangxi Medical University, P.R. China between March 2010 and April 2011. The study protocol was approved by the local Ethical Committee of the First Affiliated Hospital, Guangxi Medical University. Written informed consent to use the samples for research was obtained from the patients and clinicians. All samples were independently reviewed and diagnosed by two pathologists.

### RT-qPCR

The total RNA including miRNA was isolated from tumor sections using the miRNeasy FFPE Kit (QIAGEN, KJ Venlo, Netherlands) according to the manufacturer's instructions with modifications by changing the incubation time after mixing with proteinase K to 36 h at 55°C, meanwhile, adding proteinase K every 12 h to maintain its concentration. RNA concentration was detected by Nanodrop 2000 (Wilmington, DE 19810 USA). For *in vitro* experiments, the total RNA including miRNA was isolated as reported [27], [28]. Previously, we found the combination of RUN6B and let-7a was the most stable internal reference for HCC *in vitro* experiments by NormFinder and geNorm software, whereas the combination of RUN6B and RUN48 was the best housekeeping gene for HCC FFPE work (data not shown). Thus, different internal references were used in the current study. The primers for miR-34a, RNU6B, RNU48 and let-7a were included in TaqMan® MicroRNA Assays (4427975, Applied Biosystems, Life Technologies Grand Island, NY 14072 USA). Sequence of miRNA and references using in current study are : miR-34a (Applied Biosystems Cat. No. 4427975-000425) : UGGCAGUGUCUUAGCUGGUUGU; RNU6B (Applied Biosystems Cat. No. 4427975- 001093): CGCAAGGAUGACACGCAAAUUCGUGAAGCGUUCCAUAUUUUU; RNU48 (Applied Biosystems Cat. No. 4427975- 001006): GAUGACCCCAGGUAACUCUGAGUGUGUCGCUGAUGCCAUCACCGCAGCGCUCUGACC; let-7a (Applied Biosystems Cat. No. 4427975- 000377): UGAGGUAGUAGGUUGUAUAGUU. The reverse primers were also used in the reverse transcription step with TaqMan® MicroRNA Reverse Transcription Kit (4366596, Applied Biosystems, Life Technologies Grand Island, NY 14072 USA) in a total volume of 10 µl. Real-time qPCR for miRNA was performed with Applied Biosystems PCR7900. The miR-34a abundance in each sample was normalized to its references. The miR-34a expression in FFPE experiment was calculated with the formula 2^−Δcq^, and the change ratio of miR-34a in the *in vitro* experiments was: (1-1/2^ΔΔCq^)×100% [29].

### Re-expression and inhibition of miR-34a in HCC cells

The human HCC-derived cell lines HepB3 (HB-8064), HepG2 (HB-8065) and SNU449 (CRL-2234) were purchased from the American Type Culture Collection (ATCC, Rockville, MD,USA). They were cultured as previously described [10]. All *in vitro* experiments were performed in triplicate. HCC cells were seeded in a 24-well plate (2.5×10^4^ cells per well) or a 96-well plate (2.5×10^3^ cells per well) and incubated at 37°C for 24 h. The cells were then transfected with miR-34a inhibitor, miRNA inhibitor negative control, miR-34a mimic and miRNA mimic negative control, respectively (Ambion, Life Technologies Grand Island, NY 14072 USA) at a final concentration of 200 nmol/L for 96 h using combiMAGnetofection (OZ BIOSCIENCES, Marseille cedex 9 France) in accordance with manufacturer's procedure. The c-MET specific siRNAs as an “on target smartpool” and their scrambled controls (Thermo Scientific Dharmacon, Blenheim, England) were transfected into HCC cells with the same method as above. The sequences of the siRNAs are summarized in Table 2. A positive “TOX” control for transfection efficiency was included, which is a proprietary RNA oligonucleotide that induces cell death (Thermo Scientific Dharmacon, Blenheim, England). A c-MET kinase inhibitor, su11274 (Sigma-Aldrich N.V. Zwijndrecht, Netherlands) was prepared in dimethyl sulfoxide (DMSO) and stored at −80°C. The drug was diluted in fresh DMEM with a final concentration of DMSO less than 0.1% in all experiments.

**Table 2:** siRNAs used in the study.

Name of siRNA	sequence	Location and length(nt)	GC content(%)
c-MET siRNA1188	GAGCCAGCCTGAATGATGA	1188-1206(19)	53
Scrambled c-MET siRNA1188	GGAGCAACGAGGATTACCT	NA(19)	53
c-MET siRNA2950	GAACAGCGAGCTAAATATA	2950-2968(19)	37
Scrambled c-MET siRNA2950	GTGACACGAAACAGTATAA	NA(19)	37
c-MET siRNA3191	GTAAGTGCCCGAAGTGTAA	3191-3209(19)	47
Scrambled c-MET siRNA3191	GTAGCAAGCGACTGATGTA	NA(19)	47
c-MET siRNA4235	GAACTGGTGTCCCGGATAT	4235-4253(19)	53
Scrambled c-MET siRNA4235	GCGATAGCGGTCTGTACTA	NA(19)	53

NA: not applicable.

### Cell function detections

Cell viability, cell proliferation, apoptosis and nuclear morphology and caspase-3/7 activity were performed as described previously [27], [29], [30] to study the effects of miR-34a inhibitor and miR-34a mimic. Migration and invasion of HepG2 cells were assessed using chamber with 8 µm pore filters (6.5 mm in diameter, 8 µm pore size, Corning, USA) as described previously [14]. The migration ratio and invasion ratio were calculated as compared to the mock control [14].

### Western blot analysis

Western blot were performed as described previously [27], [29], [30]. The following primary antibodies were used: c-MET (L41G3, Cell Signaling), phospho-AKT/PKB (pS473, Invitrogen), phospho-ERK1/2 (pTpY185/187, Invitrogen), phospho-stat5 (pY694, BD Biosciences), Caspase-3 (8G10, Cell Signaling) and β-actin (Sigma-Aldrich N.V.).

### Statistical analysis

SPSS19.0 (Munich, Germany) was performed for statistical analysis. Results were representative of three independent experiments unless stated otherwise. Values were presented as the mean ± standard deviation (SD). One-way Analysis of Variance (ANOVA) test was used to analyze significance between groups. The Least Significant Difference (LSD) method of multiple comparisons with parental and control group was applied when the probability for ANOVA was statistically significant. Student's paired or unpaired *t*-test were used to analyze significance between paired or unpaired groups. Statistical significance was determined at a *P*<0.05 level. In the analysis of additivity and synergism, the theoretical zero-interaction (exactly additive) dose-response curve for each miR-34a mimic+other agent combination was calculated by applying Bliss independence criterion [17] and was also assessed by the Biosoft CalcuSyn program (Ferguson, MO, USA). The Combination Index (CI) was used to express synergism (CI<1), additive effect (CI = 1), or antagonism (CI>1) [31].

## Supporting Information

Figure S1 **Combinational effect of c-MET siRNA and miR-34a mimic in HepG2 cells.** c-MET siRNA (0.001, 0.025, 0.05, 0.1 and 0.2 µM) was combined with miR-34a mimic (0.001, 0.025, 0.05, 0.1 and 0.2 µM) and cell proliferation was detected by MTS assay. Biosoft CalcuSyn program was used to calculate (A): Median-effect plot, (B) Dose-effect curve, (C) CI. This indicates no synergistic effect, (D) Algebraic estimate.
(TIF)

Figure S2 **Combinational effect of su11274 and miR-34a mimic in HepG2 cells.** su11274 (0.001, 0.01, 0.1, 1 and 10 µM) was combined with miR-34a mimic (0.001, 0.025, 0.05, 0.1 and 0.2 µM) and cell proliferation was detected by MTS assay. Biosoft CalcuSyn program was used to calculate (A): Median-effect plot, (B) Dose-effect curve, (C) CI. This indicates no synergistic effect, (D) Algebraic estimate.
(TIF)
