# Supplementary figures and images for: Underexpression of miR-34a in Hepatocellular Carcinoma and Its Contribution towards Enhancement of Proliferating Inhibitory Effects of Agents Targeting c-MET
Source: PLoS One. 2013 Apr 10;8(4):e61054. doi: 10.1371/journal.pone.0061054 (PMC3622605; doi:10.1371/journal.pone.0061054)

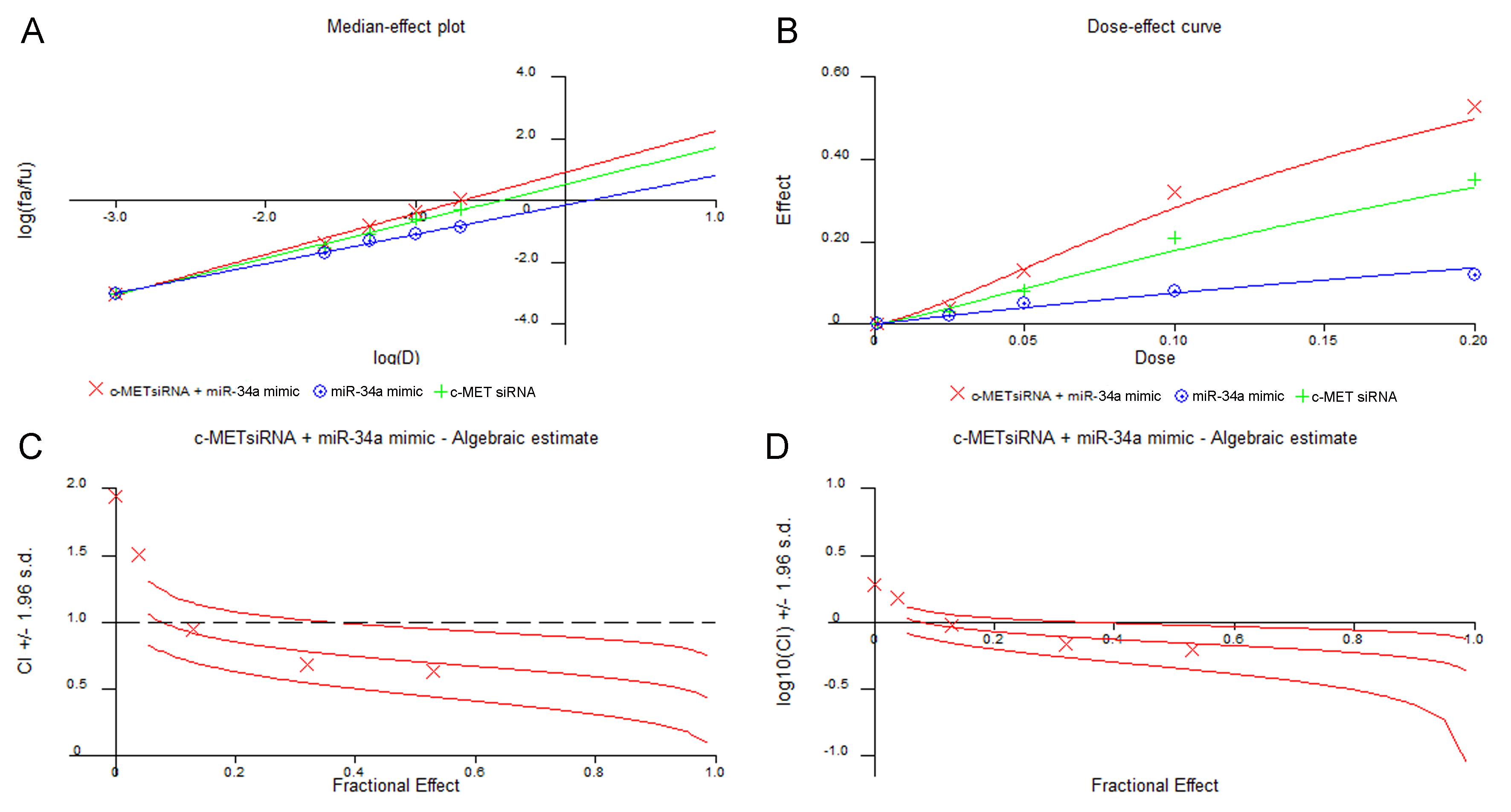

Supplement: Figure S1 — Combinational effect of c-MET siRNA and miR-34a mimic in HepG2 cells. c-MET siRNA (0.001, 0.025, 0.05, 0.1 and 0.2 µM) was combined with miR-34a mimic (0.001, 0.025, 0.05, 0.1 and 0.2 µM) and cell proliferation was detected by MTS assay. Biosoft CalcuSyn program was used to calculate (A): Median-effect plot, (B) Dose-effect curve, (C) CI. This indicates no synergistic effect, (D) Algebraic estimate. (TIF) [file pone.0061054.s001.tif]

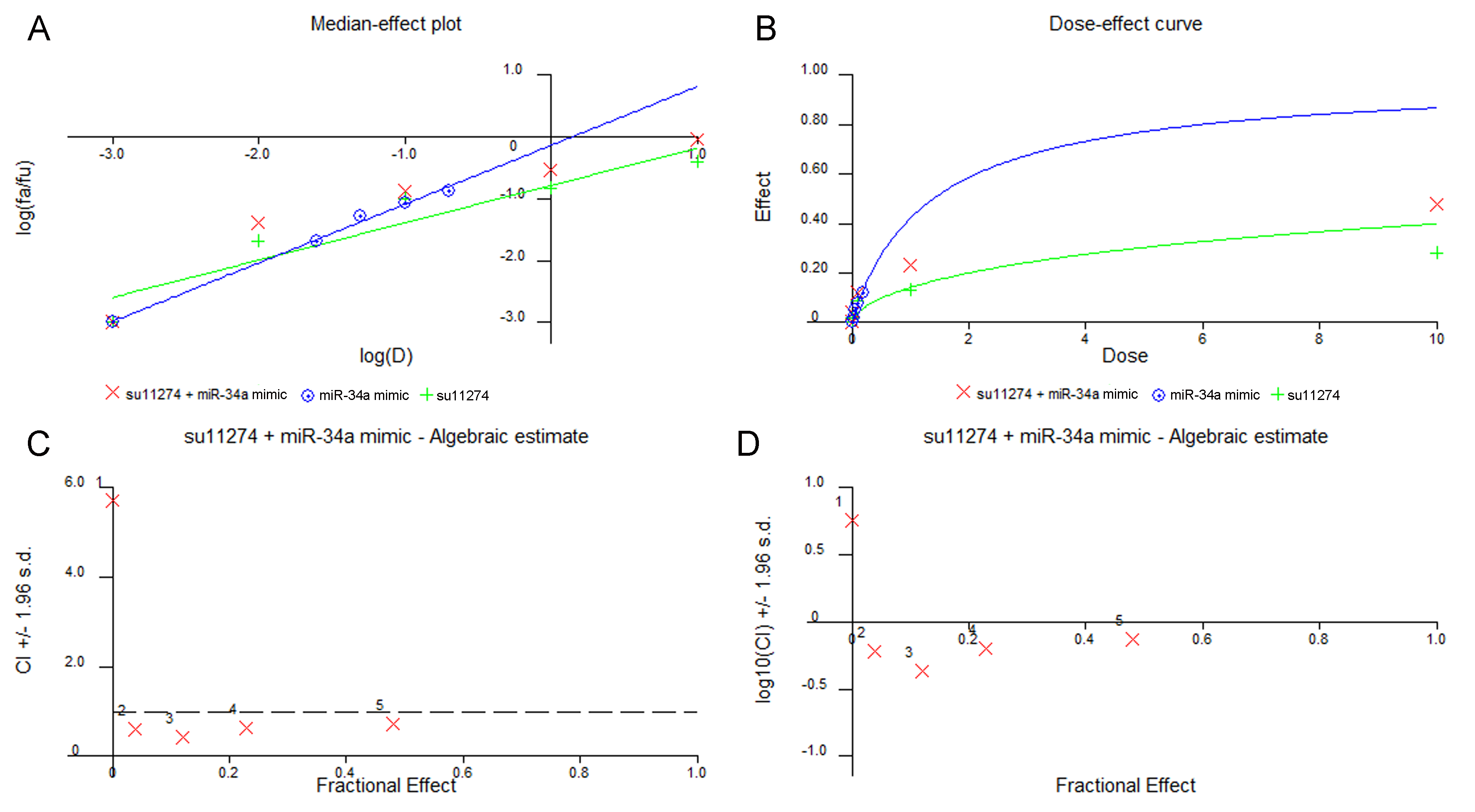

Supplement: Figure S2 — Combinational effect of su11274 and miR-34a mimic in HepG2 cells. su11274 (0.001, 0.01, 0.1, 1 and 10 µM) was combined with miR-34a mimic (0.001, 0.025, 0.05, 0.1 and 0.2 µM) and cell proliferation was detected by MTS assay. Biosoft CalcuSyn program was used to calculate (A): Median-effect plot, (B) Dose-effect curve, (C) CI. This indicates no synergistic effect, (D) Algebraic estimate. (TIF) [file pone.0061054.s002.tif]
